# Rates of Seroprotection against Vaccine-Preventable Infectious Diseases in HIV-Exposed and -Unexposed Malawian Infants

**DOI:** 10.3390/pathogens12070938

**Published:** 2023-07-14

**Authors:** Silvia Baroncelli, Clementina Maria Galluzzo, Stefano Orlando, Maria Franca Pirillo, Richard Luhanga, Robert Mphwere, Thom Kavalo, Roberta Amici, Marco Floridia, Mauro Andreotti, Fausto Ciccacci, Paola Scarcella, Maria Cristina Marazzi, Marina Giuliano

**Affiliations:** 1 National Center for Global Health, Istituto Superiore di Sanità, Viale Regina Elena 299, 00161 Rome, Italymarina.giuliano@iss.it (M.G.); 2 Department of Biomedicine and Prevention, University of Rome Tor Vergata, Via Montpellier 1, 00133 Rome, Italy; 3 DREAM Program, Community of S. Egidio, P.O. Box 30355, Blantyre 312200, Malawi; 4 UniCamillus, Saint Camillus International University of Health Sciences, Via di Sant’Alessandro 8, 00131 Rome, Italy; 5 DREAM Program, Community of S. Egidio, Piazza Sant’Egidio 3, 00153 Rome, Italy

**Keywords:** vaccine-preventable diseases, IgG protective levels, HIV in utero-exposition, *Haemophilus influenzae* type B, hepatitis B, *Streptococcus pneumoniae*, infants, Africa

## Abstract

Background: The evaluation of seroprotection rates against vaccine-preventable infectious diseases allows for the identification of risk populations. HIV-exposed infants, even if not infected with HIV, have higher morbidity and mortality in comparison to unexposed counterparts. The aim of this study was to compare the specific IgG levels against *Haemophilus influenzae* type-B (HiB), Hepatitis-B (HBV), and *Streptococcus pneumoniae* (Spn) in two groups of infants (HIV-exposed and HIV-unexposed) living in Malawi. Methods: Blood samples from 62 infants, 49 HIV-exposed, uninfected (HEU), and born to women living with HIV and 13 HIV-unexposed and uninfected (HUU), were collected at 6 months, and specific IgG levels were determined using ELISA tests. Results: The antibody levels against HiB, HBV, and Spn were similar in the two groups. At six months, all HUU infants and 81.6% of HEU infants showed seroprotective levels against HiB, while a percentage of protection varying from 80.6 to 84.6% was observed for HBV and Spn regardless of HIV exposure. Only 59.2% of HEU and 69.2% of HUU infants showed antibody protection against all three pathogens. Conclusions: These results indicate similar rates of seroprotection among HEU and HUU infants but also suggest that a consistent fraction of infants received incomplete vaccinations. Strategies to enforce participation in immunization programs in Malawi should be a health priority.

## 1. Introduction

Malawi is a sub-Saharan African country with a high HIV/AIDS prevalence [1]. In 2020, it had an estimated rate of under-five mortality of 38.6 per 1000 live births, which was mostly caused by malaria, diarrhea, and pneumonia [2]. The national HIV prevalence among childbearing women has been estimated to be around 10.3% [3], but the early adoption in 2011 of the Option B+ strategy for the prevention of mother-to-child transmission (PMTCT) [4] has limited the rate of acquisition of perinatal HIV infections, which has declined in the past few years to levels between 4 and 10% [5]. At the same time, the population of children who do not acquire the infection but are exposed to HIV and antiretroviral drugs (HIV-Exposed and Uninfected, HEU) increased significantly, exceeding 7.0% of the general child population in 2021 [3]. Although not infected with HIV, the exposed children have been reported to have a higher vulnerability to viral and bacterial respiratory tract infections, invasive pneumococcal and group B Streptococcal infections, and gastrointestinal disease [6], and they have a two- to threefold higher risk of hospitalization in comparison to unexposed (HIV-Unexposed and Uninfected, HUU) children [7,8,9]. The immunological abnormalities of HEU infants have been attributed to an impaired maternal IgG transplacental passage [10] and an incomplete B and T cell response [11], which can influence the ability to respond to vaccinations [12].

Studies comparing vaccine responses among HEU versus HUU infants have provided controversial results, showing higher, lower, or similar responses against different vaccines in various reports [13].

To determine if inadequate protection against vaccine-preventable diseases could be responsible for the higher vulnerability of the HEU population, in the present study, we aimed to analyze the rates of seroprotection against *Haemophilus influenzae* type B (HiB), Hepatitis B virus (HBV), and *Streptococcus pneumoniae* (Spn) among two contemporary cohorts of HEU and HUU infants in Malawi. Assessing the actual level of protection is important because although Malawian official reports indicate an overall vaccine coverage in the country of around 90–95% [14], field studies have revealed that in the last few years, the deterioration of immunization services and the high dropout rate have increased the number of children with an incomplete vaccination schedule and who are not fully protected from preventable diseases [15,16]. Most of these studies are based on interviews or the analysis of documentation, while specific immunological studies are lacking. 

## 2. Materials and Methods

### 2.1. Population Characteristics

The present analysis is part of a study conducted between January 2019 and June 2021 that aimed to assess the factors that determine maternal retention in programs for the prevention of vertical HIV transmission and to compare the health of HIV-exposed infants under Option B+ with that of a parallel cohort of HIV-unexposed infants [17]. The assessment of infant health included the evaluation of growth parameters, the determination of the immune responses to vaccine-preventable diseases (i.e., the aim of the present report), and the incidence of infectious and non-infectious events [18]. The study was conducted within the structures of the DREAM (Disease Relief through Excellent and Advanced Means) Program of the Community of S. Egidio, an Italian faith-based non-governmental organization delivering a range of health services in several African countries [19]. Three clinical sites, all located in the Blantyre district, were involved: the urban DREAM Center in Mandala, Blantyre, and the semi-urban sites of Chileka and Machinjiri. Mothers living with HIV (LWH) and HIV-negative mothers (>18 years of age) were enrolled during pregnancy, specifically between 32 and 36 weeks of gestation, at which point their HIV-positive (or negative) test documentation was confirmed, and demographic, clinical, and socioeconomic information was collected. Mother–child pairs of both groups were followed with monthly visits until 12 months from delivery. Among HEU infants, a polymerase chain reaction test was performed at 6 and 48 weeks for HIV-DNA detection. The vaccination schedule for anti-HiB, -HBV, and -Spn in the country includes 3 doses administered at 6, 10, and 14 weeks of age. Information about adherence to the routine immunization schedule was collected during the monthly visits. Mothers were asked to show health passports or any other document wherein the receipt of vaccinations was reported, and in their absence, their verbal responses were considered. The HiB and HBV antigens are part of the same vaccine preparation (PENTA), while the pneumococcal conjugate vaccine-13 (Pevenar13, PCV13) is scheduled at the same time but using a different preparation. 

At 6 months, a blood sample was collected from the infants by locally trained people. Plasma was separated and stored frozen locally and subsequently shipped in dry ice to the Laboratory of the Istituto Superiore di Sanità in Rome, where the samples were stored at −80 °C until the analyses were performed. All the available samples from 6-month-old infants were analyzed in the present serological study.

### 2.2. Laboratory Evaluations

The IgG levels against *H. influenzae* and *Streptococcus pneumoniae* were evaluated using commercial ELISA kits (VaccZyme™ Haemophilus influenzae type B IgG kit, and VaccZymeTM anti-PCP IgG Enzyme immune Assay, Binding Site, Birmingham, UK) according to the manufacturer’s instructions. The range of detection for anti-HiB IgG was 0.11–9.0 mg/L. An antibody level greater than 0.15 mg/L is considered the minimum protective level, but 1.0 mg/L is considered the optimal IgG level for long-term protection [20]. 

The range of detection for anti-Spn IgG was 3.3–270 mg/L. There is no international agreement on the anti-Spn antibody protective level; a surrogate level of protection of ≥50 mg/L is generally accepted [20,21] based on the results of previous studies (using the same immunoassays on healthy, unvaccinated subjects). To verify that the antibody protective level, extrapolated from European patients, could be applied to an African infant population, we assessed, using the same commercial assay, the anti-Spn antibody levels in 69 samples obtained from 6-month-old HEU Malawian infants enrolled in a previous study conducted between 2008 and 2010 (before the introduction of the PCV13 vaccination program) [22]. Given the mean antibody level of the negative samples (6.95 mg/L), the value of 3SD (3 × 11.4), and the final value of 41.1 mg/L, the protection level for anti-Spn IgG of 50 mg/L was considered reasonable. 

Anti-hepatitis B surface antigen (HBs) IgG levels were evaluated using Monolisa Anti-HBs Plus EIA kits (Bio-Rad Laboratories, Marnes La Coquette, France). The range of detection was between 2.0 and 1000 mIU/mL. The minimum protective level has been established at 10 mIU/mL, and levels >100 mIU/mL are considered necessary for long-term protection against infection [23]. 

### 2.3. Statistical Analysis

SPSS software, version 27 (IBM, Somers, NY, USA), was used for statistical analyses. Socio-demographic data are presented as medians with interquartile ranges (IQR) and percentages. Geometric mean and 95% CI were used to assess antibody levels. The lower and upper levels of the range of detection of the different ELISA kits were used to categorize undetectable or “above the curve” results for the statistical analysis. Differences between groups were evaluated using the χ^2^ test or Fisher’s exact test when appropriate for categorical variables and using the Mann–Whitney U test for quantitative variables. Spearman’s correlation coefficient was used to evaluate correlations between quantitative variables. Differences were considered statistically significant at the following level: *p* < 0.05. 

## 3. Results

### 3.1. Population Characteristics

The study aimed to enroll 150 women living with HIV along with their infants and 150 HIV-negative women along with their infants; however, difficulties were encountered in enrolling HIV-negative women (a total number of 73 was reached), and a high rate of loss to follow-up (especially in HIV-negative women) occurred. Further, the study period partially overlapped the COVID-19 pandemic, which contributed to the lower-than-expected attendance in the follow-up visits (and with respect to the collection of blood samples). 

Therefore, only 62 (13 HUU and 49 HEU) infants who had 6-month samples available could be included in this study. Upon enrollment, all the women living with HIV were receiving antiretroviral treatment (mostly tenofovir + lamivudine plus efavirenz or dolutegravir). Almost half of the women (49.0%) had been receiving ART for a median of 7.1 years, while the other 51% had an ART history of less than 1 year. No difference in the mothers’ characteristics or socio-economic parameters was observed between the two groups (Table 1). Delivery occurred via the vaginal route in most cases. Median weight at post-birth visit (within 15 days from delivery) was similar in the two infant groups (HEU: 3.50 kg; HUU: 3.45 kg, *p* = 0.651). All birth weights were >2.5 kg. The growth rate did not differ between infants born to HIV-negative mothers and those born to mothers living with HIV, showing similar weight gain during the first 6 months of life (Table 1). 

### 3.2. Seroprotection Rates

*Haemophilus influenzae type B*: The geometric mean concentrations (GMCs) of anti-HiB IgG were 1.39 mg/L in HEU infants and 2.54 mg/L in HUU infants (*p* = 0.194) (Table 2). The proportion of infants with the minimum protective level (>0.15 mg/L) was 81.6% (40/49) in the HEU infants and 100% (13/13) in the HUU infants (*p* = 0.095). The proportion of infants with anti HiB levels > 1.0 mg/L did not differ between the two groups: 65.3% in the HEU infants and 76.9% in the HUU infants (*p* = 0.426). 

*Hepatitis B*: Anti-HBs IgG levels did not significantly differ between the two groups of infants (HEU: 62.5 mIU/mL, HUU: 74.7 mIU/mL, *p* = 0.949, Table 2). Overall, 81.6% of the HEU infants and 84.6% of the HUU infants had anti HBs-IgG protective (≥10 mUI/mL) levels, and there was no significant difference between the groups (*p* = 0.802). Only 53.1% of the HEU infants and 61.5% of the HUU infants (*p* = 0.756) had anti-HBs IgG levels higher than 100 mIU/mL. 

*Streptococcus pneumoniae*: No significant differences were observed in the anti-Spn IgG levels between the HEU and HUU infants (83.7 mg/L vs. 90.9 mg/L, respectively, *p* = 0.809, Table 2). The proportion of infants with a level of anti-Spn IgG > 50 mg/L did not differ between the HEU and HUU groups (HEU: 80.9%, HUU: 84.6%, *p* = 0.576). 

### 3.3. Vaccination Documentation 

Vaccines were not administered in the same clinical health centers the mothers and infants had visited. The mothers were asked to show their health passports during the scheduled visits (months 2, 3, and 4) following the first, second, and third dose of the vaccines. If written documents were missing, their verbal reports were recorded. Full compliance with all three visits was achieved in 30/49 (62.1%) of the mothers LWH and 7/13 (53.8%) of the HIV-negative mothers. Proportions of 38.8% and 46.2% of the mothers of the two groups, respectively, had missed at least one of these visits, resulting in an incomplete verification of the vaccination statuses of their infants. However, no significant correlation between the number of missed visits and the infants’ seroprotective status against vaccine antigens was found (although the low number of infants should be considered). 

### 3.4. Analyses of Seroprotection Levels

**Figure 1:**
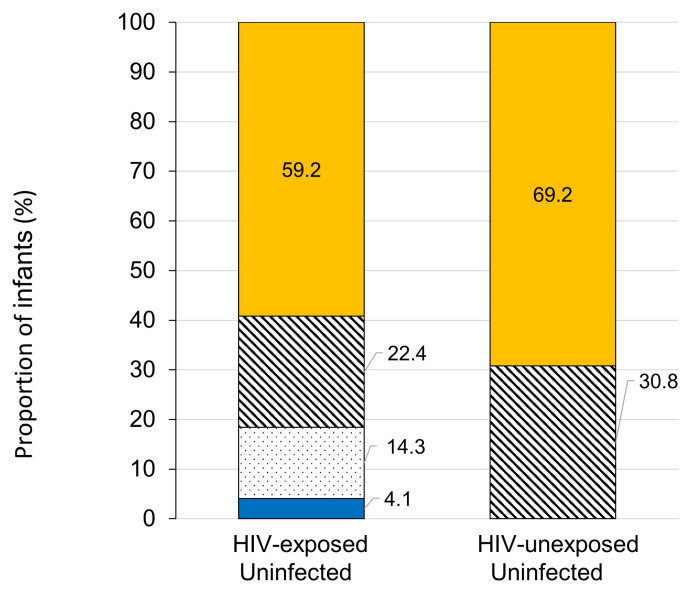
Rates of infants with protective antibody levels (HiB > 0.15 mg/L, HBV > 10.0 mUI/mL, and *S. pneumoniae* ≥ 50 mg/L) against the three pathogens among HEU and HUU groups. Blue: No protective levels against any pathogen. Dotted: Protective levels against only 1 pathogen. Diagonal stripes: Protective levels against 2 out of 3 pathogens. Yellow: Protective levels against all 3 pathogens.

Protective levels against all three pathogens were observed in only 61.3% (38/62) of the infants, 59.2% of the HEU infants, and 69.2% of the HUU infants (*p* = 0.415, Figure 1). In the HUU group, protective levels against at least two out of three pathogens were observed in 30.8% of the HUU infants versus 22.4% in the HEU group. Seven (14.3%) HEU infants had protective levels against only one pathogen, and two infants (4.1%) did not show evidence of protection against any of the three analyzed vaccine-preventable diseases.

The antibody concentrations were not correlated with either maternal age (HiB: r = 0.240, *p* = 0.854; HBV: r = 0.23, *p* = 0.861; PCP: r = 0.123, *p* = 0.353) or the ART duration of mothers living with HIV (Hib: r = 0.110, *p* = 0.468; HBV: r = 0.230, *p* = 0.833; PCP: r = 0.124, *p* = 0.411). No sex-related differences or correlations between the magnitude of the IgG response and infants’ weight were detected. The analysis of socio-demographic parameters (maternal education level, maternal employment, and residence) did not reveal any significant associations with antibody concentrations. 

The study timeline overlapped with the spread of COVID-19 in the country. Among the 62 infants, most of them (79.0%, HEU:39, HUU:9) were scheduled to complete their vaccination programs in the pre-COVID era, and 14 infants (22.6%, HEU:10, HUU:4) were scheduled to start their vaccination programs after the promulgation of the containment strategies. Overall, the rates of infants with protective levels against all three pathogens were similar in the pre- and post-COVID-19 periods (60.4% vs. 64.3%, *p* = 0.381), with no differences between the groups.

## 4. Discussion

In terms of the assessment of the antibody concentrations of HEU infants, the results of this study show seroprotection rates against vaccine-preventable diseases that are similar to those of their unexposed counterparts. However, the serological analysis highlighted high rates of 6-month-old infants without IgG protective levels against the three analyzed relevant infectious diseases.

All the HUU infants and 81.6% of the HEU infants showed seroprotective levels against *H. influenzae*, while the proportions of infants with antibody protective levels against Hepatitis B and *S. pneumoniae* varied from 80.6 to 84.6%, without statistically significant differences between the two groups. Our data seem to be in agreement with other serological studies that did not find significant differences between HEU and HUU populations, indicating that vaccine-preventable diseases lead to the development of specific antibody responses that are equivalent in HEU and HUU infants [24,25,26,27]. However, in the present study, the levels of protection presented by the HUU children were consistently slightly higher than those presented by the HEU children, and we cannot exclude the possibility that the lack of differences could be related to the reduced statistical power due to the limited sample size. 

Using the minimum protective levels, roughly 20% of the infants at 6 months of age did not show protective levels against at least one of the three pathogens, and only 59.2% of the HEU infants and 69.2% of the HUU infants showed protective levels against all three pathogens studied. 

In 2021, the WHO/UNICEF estimated an immunization coverage of 93% for the HBV, HiB, and Spn vaccines in Malawian infants [14], which is far above the prevalence of protective responses that we found in our study. Indeed, the seroprotection rates that we found are consistent with the findings of a recent study that, using the third dose of the diphtheria–tetanus–pertussis vaccine (DPT3) as a proxy indicator for immunization performance, reported that among the 25 sub-Saharan African countries, full vaccination coverage ranged from 24% in Guinea to 93% in Rwanda [28]. In particular, in Malawi, between 2010 and 2015, the proportion of preschool-aged children who had been fully vaccinated against preventable diseases was between 76% and 81% [29]. After almost a decade, our serological analysis shows a similar scenario, confirming low rates of seroprotection against vaccine-preventable diseases in Malawian infants.

No determinants of missing a vaccination (maternal age, levels of education, or socioeconomic situation), which in other studies [29,30] have been associated with not being vaccinated, could be identified in this study, but the low proportion of infants with antibody levels > 1.0 mg/L for anti-Hib and 100 mIU/mL for anti-HBs is indicative of long-term protection and supports the hypothesis of an incomplete vaccination program with one or more missing vaccine doses. 

In Malawi, the services providing immunization are distinct from the health facilities, and the registration of vaccinations in children’s health passports, although officially planned, is not common in reality [16,31]. A health passport is generally provided to mothers after their infant(s)’s birth, but this document has been reported to have limited reliability, which is related to different factors, varying from the lack of training of the health operators to the limited understanding of its meaning and importance among the mothers [29,32]. Also, in this study, roughly 42.0% of mothers missed at least one of the three scheduled visits following the administration of the vaccine doses, making it even more difficult to verify adherence to the immunization program. 

It is also important to underline (and to ascribe as the main limitation of this study) that the study design included the verification of health passports (or of other written documents) to measure the adherence to the vaccine schedule, but in the absence of written documents, maternal verbal reports were also considered. However, the mode of verification of attendance at immunization visits was not recorded. 

Our study period mostly overlapped with the first wave of the COVID-19 pandemic. The health policies for COVID-19 containment in Malawi, implemented at the end of March 2020 [33], could have worsened the rate of missed vaccinations. However, the temporal analysis that we performed did not show a significant difference in the proportion of infants with protective levels between the pre- and post-pandemic periods. 

This study has important limitations, one of which, as already mentioned, is that the adherence to the immunization schedule was not strictly based on official documentation, and data from verbal sources tend to overestimate the true degree of coverage [34]. Another important limitation is the limited sample size, particularly for the HUU infant group, which reduced the statistical power with which to detect differences in the seroprotection rates between the study groups. In a previous analysis of the same cohort, we reported a high dropout rate among HIV-negative women compared to HIV-positive women [18]. We hypothesized that while the mothers living with HIV were more motivated to maintain tighter contact with the healthcare services to receive regular HIV treatments [35,36], this was not true for the HIV-negative women and their HUU infants. Nevertheless, we believe that this study can offer a direct representation of the seroprotection rates against vaccine-preventable diseases in and around Blantyre during and soon after the COVID-19 pandemic period and highlights the overwhelming difficulties in the assessment of the true prevalence of non- and under-vaccinated children in Malawi only based on the available documentation.

## 5. Conclusions

In conclusion, our study provides reassuring data on the immunological status of HEU infants, indicating limited immunological differences between HIV-exposed and unexposed infants, but reveals high rates of HEU and HUU infants without antibody protective levels against vaccine-preventable diseases. Future serological studies should be carried out in combination with documentary investigations (e.g., the acquisition of reliable records of vaccination among children) in order to identify and possibly remove the main barriers contributing to the suboptimal protection levels observed in Malawi.

## Figures and Tables

**Table 1:** Infants and maternal characteristics. Values are expressed as medians and IQRs or percentages.

	HEU Infants	HUU Infants	*p* Value
Number of infants	49	13	
Sex ratio (M/F) (%)	30/19(61.2/38.8)	4/9 (30.8/69.2)	0.065
Weight at birth (kg)	3.50 (3.1–4.0)	3.45 (3.0–3.8)	0.651
Weight gain over 1–6 months (kg)	2.95 (2.48–3.43)	2.80 (2.40–3.70)	0.775
Maternal age (years)	30.0 (23.3–33.8)	30.0 (25.5–32.5)	0.994
Maternal BMI	25.0 (22.5–28.8)	26.3 (22.5–28.8)	0.970
ART duration at enrolment among mothers living with HIV (years)	0.7 (0.4–7.1)	-	-
Women with <1 year of ART (n, %)	25 (51.0%)	-	
Women with >1 year of ART (n, %)	24 (49.0%)	-	
Electricity at home, n, (%)	18 (37.5%)	4 (30.8%)	0.654
Water at home, n, (%)	28 (58.3%)	10 (76.9%)	0.220
Residency			0.176
Urban	12 (24.5%)	1 (7.7%)	
Semirural/rural	36 (75.0.1%)	12 (92.3%)	
Maternal Education			0.835
Primary or No Education	28 (58.3%)	8 (61.5%)	
Secondary or higher	20 (40.8%)	5 (38.5%)	

**Table 2:** Antibody levels against Haemophilus influenzae type B (anti-HiB), Hepatitis B (anti-HBs), and Streptococcus pneumoniae (anti-Spn) measured at 6 months of age in 49 HIV-exposed uninfected (HEU) and 13 HIV-unexposed uninfected (HUU) infants. Values are expressed as geometric means and 95% CIs. The proportions of infants with protective levels are reported in brackets.

		All	HIV-Exposed Uninfected (HEU)	HIV-Unexposed Uninfected (HUU)	*p* Values
No. infants		62	49	13	
Anti-HiB IgG (mg/L)		1.58 (1.42–1.74)	1.39 (0.75–1.21)	2.54 (2.19–2.90)	0.194
Infants with anti-HiB IgG (n, %)	>0.15 mg/mL	53 (85.5%)	40 (81.6%)	13 (100%)	0.095
	>1.00 mg/mL	42 (67.7%)	32 (65.3%)	10 (76.9%)	0.426
Anti-HBs IgG (mIU/L)		64.9 (62.4–65.8)	62.5 (57.02–58.4)	74.7 (74.2–75.2)	0.949
Infants with anti-HBs IgG (n, %)	>10 mIU/L	51 (82.3%)	40 (81.6%)	11 (84.6%)	0.802
	>100 mIU/L	34 (54.8%)	26 (53.1%)	8 (61.5%)	0.756
Anti-Spn IgG (mg/L)		85.1 (84.7–86.9)	83.7 (71.6–73.8)	90.9 (89.5–92.4)	0.809
Infants with anti-Spn IgG (n, %)	>50 mg/L	49 (81.7%)	38 (80.9%)	11 (84.6%)	0.576

## Data Availability

The datasets analyzed in the current study are available from the corresponding author upon reasonable request.
